# The Effect of Post Heat Treatment on the Microstructure and Mechanical Properties of Cold-Sprayed Zn-6Cu Deposits

**DOI:** 10.3390/ma17246096

**Published:** 2024-12-13

**Authors:** Xiao-Zhen Hu, Xiao-Bo Tan, Bin Xie, Hai-Long Yao, Chao Yang, Tao Zhou

**Affiliations:** 1 School of Architecture Engineering and Planning, Jiujiang University, Jiujiang 332005, China; hxz5566@126.com (X.-Z.H.); nancy0793@163.com (B.X.); 2 Intelligent Manufacturing Industry College, Jiangxi University of Engineering, Xinyu 338004, China; andytxbo@163.com; 3 Jiangxi Provincial Key Laboratory of Materials Surface Engineering, School of Materials Science and Engineering, Jiujiang University, Jiujiang 332005, China; summersquid@163.com (C.Y.); 18979957787@163.com (T.Z.)

**Keywords:** cold spraying, Zn-Cu alloy, tensile property, wear behavior

## Abstract

To explore the feasibility of preparing Zn alloy bulk, Zn-6Cu deposit was prepared by cold-spraying additive manufacturing. Microstructure, tensile and wear behavior were investigated before and after heat treatment. Cold-sprayed Zn-6Cu deposit was constituted by irregular flattening particles and pores after heat treatment. Zn-6Cu deposits were composed of Zn and CuZn_5_ phases in addition to ZnO phase regardless of heat treatment, but the full width at half maximum of both the CuZn_5_ and the Zn phase were varied. The yield strength and ultimate tensile strength of Zn-6Cu deposits after post heat treatment were, respectively, increased from 83.8 ± 28.7 MPa and 159.6 ± 44.5 MPa to 89.4 ± 24.4 MPa and 223.8 ± 37.1 MPa. Fracture morphology after tensile testing exhibited main features of dimples, pores and cleaving particles. The friction coefficient and wear rate of Zn-6Cu deposits were increased after heat treatment, and the corrosive wear exhibited a lower friction coefficient and wear rate than the dry wear due to the lubricant of simulated body fluid. Grooves and localized delamination were the main wear features of Zn-6Cu deposits regardless of both the heat treatment and wear condition. This result indicates a potential application of cold-sprayed Zn-6Cu deposits comparable to the casting ones.

## 1. Introduction

Biodegradable metals have gained much attention as load-bearing biodegradable implants due to their superior mechanical properties compared with polymeric materials. Mg, Zn, and Fe are typical biodegradable metals and have become research hotspots in the field of artificial implants [1]. Zn-based alloys exhibit a lower corrosion rate than Mg alloys and a higher corrosion rate than Fe alloys [2]. Recently, Zn and its alloys have drawn increasing attention as biodegradable metals due to their superior mechanical properties, moderate degradation rate, and proper cytocompatibility [3]. However, pure Zn exhibits poor strength and ductility, which has to be improved to meet the clinical requirements.

Alloying is a general method to improve the mechanical properties of pure Zn. Different Zn alloys are explored for implant materials and show higher yield strengths and ductility than pure Zn, such as Zn-Mg [4], Zn-Li [5], Zn-Mn [6], Zn-Ag [7], Zn-Ca [8], Zn-Fe [9], Zn-Ti [10] and Zn-Cu [11]. Among these elements, Cu has a broad spectrum of antibacterial properties [12,13], and also presents anti-inflammatory, anti-microbial, and anti-proliferation properties in both its metallic form and chemical compound form [14,15]. Zn-Cu alloys not only improve the mechanical properties of pure Zn, but also exhibit antibacterial properties and biocompatibility. In most studies, Zn-Cu alloys are usually prepared by casting [16]. Although alloying elements could improve the mechanical properties of pure Zn, large grains and dendrite secondary phases hinder their further improvements. It is reported that combining homogenization with plastic deformation is widely applied to decrease grain size and significantly improve yield strengths and elongations of Zn-Cu alloys [16]. After homogenization at high temperature, some secondary phases are dissolved and grains grow. Dendrite secondary phases and large grains in Zn-Cu alloys are crushed by different plastic deformation processes, such as hot extrusion [3], equal channel angular pressing [17], high-pressure torsion [18], hot rolling [19], cold rolling [20], hot rolling followed by cold rolling [21], and hot extrusion followed by cold rolling [22]. According to classical strengthening theory, the main strengthening mechanism in metallic materials includes secondary phase strengthening, fine grain strengthening, solid solution strengthening, dislocation strengthening, and texture strengthening [23,24,25,26,27]. Therefore, combining casting with plastic deformation is a common strategy for Zn-based alloys.

Compared with casting, additive manufacturing is a feasible method to prepare different metal parts. Some studies also report that Zn parts are prepared by air plasma spraying [28] and laser additive manufacture [29,30,31]. Both laser additive manufacture and air plasma spraying are high-energy processes. However, Zn presents low melting and boiling points. It is reported that excessive vapor formation and material ejection of Zn powders result in pores and poor inter-particle bonding in the Zn parts [28]. It is difficult to gain dense Zn parts in laser additive manufacture although optimizing different process parameters [29,30,31]. A low laser energy causes irregular pores due to the lack of fusion, but an excessive laser energy causes spherical pores due to gas entrapment [32]. Additive-manufactured Zn parts could present lower mechanical properties than that by combining casting with plastic deformation, which may be mainly attributed to pores and uncontrollable grain sizes.

Cold spraying is a method of low-energy and solid-state additive manufacturing technology to prepare pore and dense metallic coatings and parts [33,34]. Metal particles do not melt during cold spraying, which avoids the oxidation and the vaporization of metal powders with low melting and boiling points. Many studies report the applications of metal bulks prepared by cold-spraying additive manufacture, such as Al alloys [35], Ti alloys [36], and Cu alloys [37]. Depending on severe plastic deformations of metal particles [38], grain sizes of cold-sprayed metals are usually decreased. However, the main inter-particle bonding within deposits is the mechanical interlocking [39,40], and some metallurgic bonding could be observed in metal deposits with low melting points due to impact-induced melting [41]. Post spray heat treatment effectively improved metallurgical inter-particle diffusion, homogenization of microstructure, and in some cases reduction in porosity [36]. It is reported that cold-sprayed Cu alloys after heat treatment exhibit decreases in the yield strength and improved ductility [42,43]. However, cold-sprayed Inconel 718 alloy after heat treatment shows comparable mechanical strengths and ductility with the bulk materials and is higher than the counterpart prepared by selective laser melting after appropriate heat treatment [44], and the cold-sprayed Ti6Al4V deposit after heat treatment also improves the tensile strength [36]. It is reported that most casting Zn alloys are usually homogenized at high temperature before plastic deformations [16]. Cold-sprayed Zn alloys after heat treatment are not only homogenized, but also exhibit high inter-particle bonding and mechanical properties. Therefore, it is possible to prepare Zn-Cu parts with proper microstructure and mechanical property by cold-spraying additive manufacture after heat treatment.

In this study, Zn-6Cu deposit was prepared by cold-spraying additive manufacturing technology. The effect of post heat treatment on phase, microstructure, tensile and wear property was investigated. Wear behavior was studied by comparing dry wear with corrosive wear.

## 2. Experimental Procedure

### 2.1. Materials

**Figure 1:**
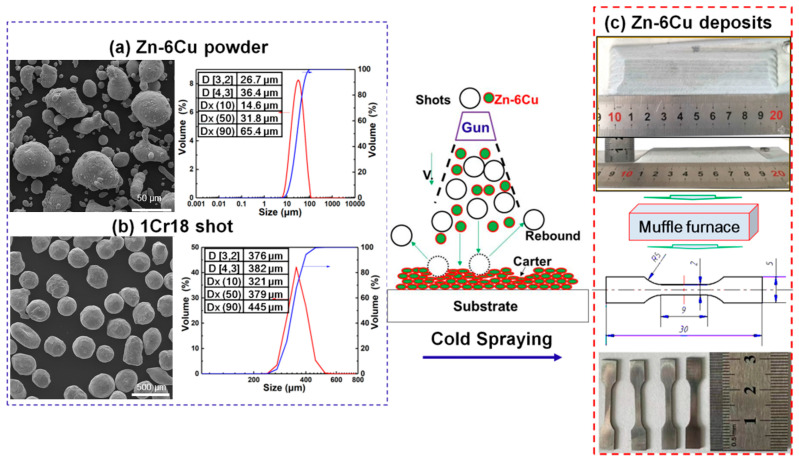
Schematic procedure of Zn-6Cu deposits prepared by cold-spraying additive manufacture. (**a**) Zn-6Cu powder, (**b**) 1Cr18 powder, (**c**) and preparation of Zn-6Cu deposits.

Commercial Zn-6Cu alloy powder (94.3 wt.% Zn and 5.7 wt.% Cu, Beijing Youxinlian Nonferrous Metals Co., Ltd., Beijing, China) and 1Cr18 stainless steel (72.63 wt.% Fe, 17.32 wt.% Cr, 5.15 wt.% Si, 4.9 wt.% Ni, SUS 304, Wuxi Weiguang Peening Materials Co., Ltd., Wuxi, China) powder were used as the original material and in situ microforging shots, respectively. Figure 1a,b shows the microstructure and the size distribution of both the Zn-6Cu powder and the 1Cr18 shot. The D10, D50 and D90 values for the Zn-6Cu powder are 14.6 μm, 31.8 μm and 65.4 μm, respectively. The D10, D50 and D90 values for the 1Cr18 shot are 321 μm, 379 μm and 445 μm, respectively. The feedstock was a powder mixture of both the Zn-6Cu powder and the 1Cr18 shot. Powder mixtures were sealed in a stainless steel vessel and then admixed using a drum mixer for 12 h at a rotation speed of 20 rpm. In the feedstock, there was 70 vol.% of Zn-6Cu powder and 30 vol.% of 1Cr18 shot. Commercial pure Al plates were cut into 100 mm × 50 mm × 6 mm pieces and are used as substrates. Before spray, Al substrates were sandblasted with 24 grit alumina by compressed air using 0.6 MPa pressure and then were ultra-sonic cleaned in ethanol for 10 min and dried by N_2_ gas.

### 2.2. Preparation of Zn-6Cu Deposit

The feedstock was deposited on the Al substrate by a cold-spraying system assembled by Xi’an Jiaotong University. A cold spray torch is equipped with a convergent–divergent Laval nozzle, which has a throat diameter of 2.7 mm, an outlet diameter of 6 mm and a convergent section length of 30 mm. The distance between the throat and the exit is 105 mm. N_2_ gas was used as the primary gas and the carrier gas. The primary gas pressure and temperature were 2.0 ± 0.1 MPa and 260 ± 20 °C, respectively. A zig-zag scan strategy was used with a step size of 2 mm. The stand-off distance and transverse speed of the torch were 20 mm and 1 mm/s, respectively. The cold-sprayed Zn-6Cu deposit with an approximate geometry (length × width × high: 100 × 20 × 10 mm) was produced by spraying multiple passes, as shown in Figure 1c. The particle velocity of Zn-6Cu powder and stainless steel shots was characterized by using an in-flight diagnostic system (DPV 2000, Tecnar Automation, Montreal, QC, Canada). This system is based on optical pyrometer and time-of-flight measurements that allow on-line measurements of the particle velocity in the spray jet. The particle detector was placed at the same spray distance.

### 2.3. Post Heat Treatment

For post heat treatment, the annealing temperature and time referred to the homogenization conditions of cast Zn-based alloys [4]. In this study, Zn-6Cu samples were heated in a muffle furnace as shown in Figure 1c. Type-A refers to that the deposit was heated at 200 °C for 4 h. Type-B refers to that the deposit was heated at 300 °C for 24 h and quenched in water. Type-C refers to that the deposit was heated at 300 °C for 24 h and quenched in water, followed by heating at 80 °C for 12 h.

After post heat treatment, the Al substrate was removed by electrical discharge machining to obtain free-standing Zn-6Cu specimens. According to DIN EN ISO 6892-1 [45], microflat tensile (MFT) tests were applied to gain direct information about the mechanical properties under the uni-axial stress state. For the preparation of MFT samples, the Zn-6Cu deposit was cut into desired test sample geometries (L × W × H: 30 × 5 × 0.5 mm; gauge length 9 mm) by electro-discharge erosion as shown in Figure 1c. Microstructure of different Zn-6Cu deposits was characterized by a scanning electron microscope (SEM, VEGA II, Tescan, Brno, Czech Republic). The porosity was measured by a photoshop software (Adobe Photoshop 7.0) through using at least five polished cross-section images of 500× for each condition. Phase structures of different Zn-6Cu samples were identified by X-ray diffraction (XRD, D8 Advance, Bruker, Karlsruhe, Germany) at CuKα radiation of 1.5418 Å, an operating voltage of 35 kV, operating current of 35 mA, 2θ range of 30–90°, and a scan rate of 0.1°/s.

### 2.4. Tensile Testing

In deviation from the standard for MFT testing, the experiments were performed using a load controlled procedure with a fixed strain rate of 0.00025 1/s. The elongation was optically recorded by a CCD camera of type videoXtens and analyzed by the commercially available software testXpert III, both from Zwick/Roell (GmbH & Co., Ulm, Germany). At least five samples was tested for each condition. Microhardness of Zn-6Cu deposits before and after post heat treatment was obtained by Vickers microhardness tester (HVS-1000, Shanghai, China) at a load of 25 gf and a dwell time of 20 s. At least ten indents was detected for each condition. The fracture morphology of Zn-6Cu samples after microflat tensile testing was characterized by a scanning electron microscope.

### 2.5. Wear Testing

The friction and wear behavior of the as-sprayed, Type-A, Type-B, and Type-C deposits were investigated using a reciprocating friction tester (HSR-2M; Lanzhou Zhongke Kaihua Technology Development Co., Lanzhou, China). The sample size for wear testing was 15 × 15 × 2 mm. At least five samples was used for each condition. The tests were carried out under a wear load of 10N, a friction stroke length of 5 mm, a friction frequency of 5 Hz, a testing time of 30 min, and a friction pair of Si_3_N_4_ with a diameter of 4 mm. The sample surfaces were polished to a roughness (Ra) of 0.5 μm using diamond paste before wear testing. Dry wear tests were performed at room temperature. Corrosive wear tests were carried out by immersing the samples in a sink containing 50 mL stimulated body fluid (SBF, Qingdao Jisskang Biotechnology Co., Ltd., Qingdao, China) of 37 °C. The SBF contains 142.0 mM Na^+^, 5.0 mM K^+^, 1.5 mM Mg^2+^, 2.5 mM Ca^2+^, 103.0 mK Cl^−^, 10.0 mM HCO_3_^−^, 1.0 mM HPO_4_^2−^, 0.5 mM SO_4_^2−^. After wear test, the samples were washed in distilled water. The worn surface morphology was characterized by SEM with an energy dispersive x-ray analysis. The worn width, depth and profiles were measured by a profilometer equipped on the friction tester.

## 3. Results and Discussion

### 3.1. Phase of Zn-6Cu Deposits Before and After Heat Treatment

**Figure 2:**
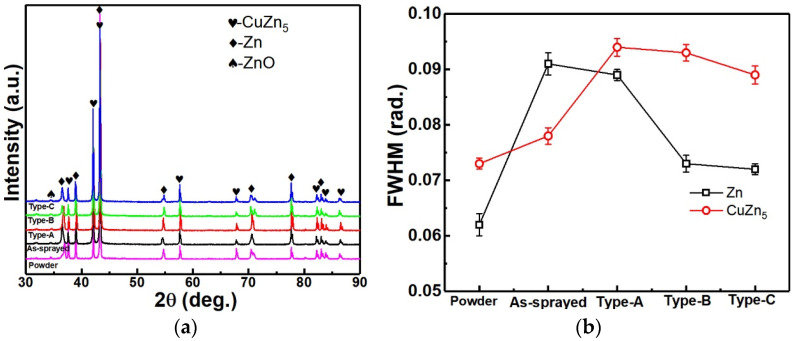
XRD patterns of Zn-6Cu deposits before and after heat treatment. (**a**) XRD patterns, (**b**) FWHM.

Figure 2 shows that Zn-6Cu deposits before and after heat treatment were composed of Zn and CuZn_5_ in addition to a little ZnO. It can be found that the full width at half maximum (FWHM) of both the CuZn_5_ phase (2θ = 42.06°) and the Zn phase (2θ = 43.28°) were increased after cold spraying. However, the FWHM of Zn phase was decreased and the CuZn_5_ phase was increased after post heat treatment. It is reported that the grain size, the stacking fault, the structural disorder, and the stress could result in the variation in FWHM in cold-sprayed metals [46]. The increase in the FWHM was mainly attributed to the serious plastic deformation of Zn-6Cu particles during high velocity impact [47]. After post heat treatment, the stacking fault, the structural disorder and the stress could be reduced or eliminated. This indicates that the Zn grains grew and the CuZn_5_ grains refined after heat treatment. The gain growth is a common phenomenon in cold-sprayed metal deposits during post heat treatment [48,49]. The refinement of second phase could be attributed to the solid solution, which is usually reported in Zn-Cu alloy after heat treatment [50]. Therefore, it can be said that the grains of Zn-6Cu deposits were refined by cold spraying deposition, while post heat treatment exhibited different effects on the Zn and the CuZn_5_ phases.

### 3.2. Microstructure of Zn-6Cu Deposits Before and After Heat Treatment

**Figure 3:**
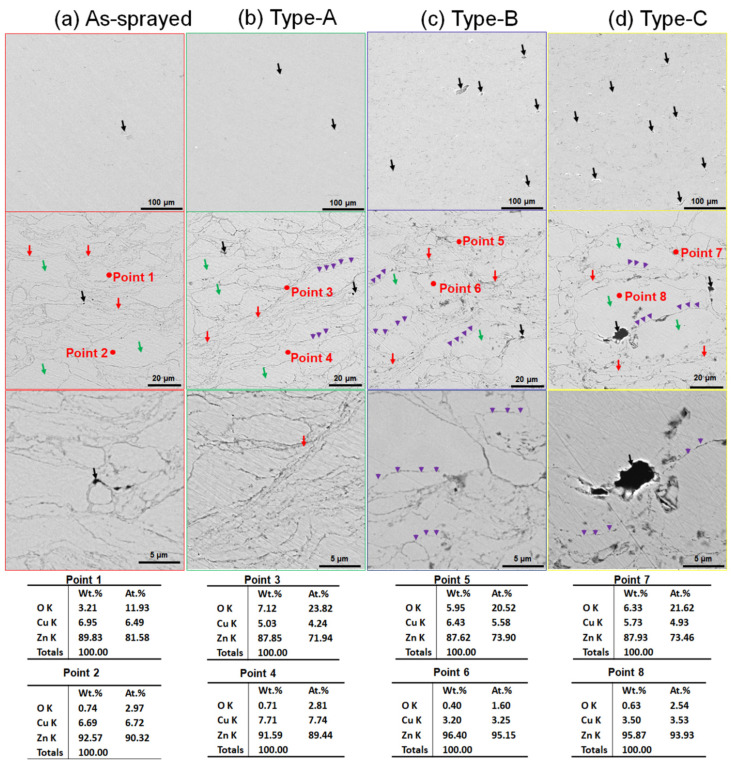
Microstructures of Zn-6Cu deposits before and after heat treatment. Black arrows: pores. Red arrows: strips. Green arrows: flattening particles. Purple arrows: coalesced.

Figure 3 shows microstructure of cold-sprayed Zn-Cu deposits before and after heat treatment. During the deposition process, both Zn-Cu particles and stainless steel shots impact onto the substrate and the deposited particles. The in-flight velocity of Zn-6Cu particles was approximately 395 ± 53 m/s, and the stainless steel shot was approximately 235 ± 63 m/s. The high velocity impact of Zn-6Cu particles exhibited severe plastic deformations. Furthermore, stainless steel shots impacted onto the deposited Zn-6Cu particles, which enhanced plastic deformations of the Zn-6Cu particles in deposits [47,51]. However, the stainless steel shots rebound from the deposit due to their large sizes and low particle velocities. No stainless steel shots were observed in Zn-6Cu deposits as shown in Figure 3. This results in the deposition efficiency of the Zn-6Cu deposit approximately 42%. The Zn-6Cu particles exhibited severe plastic deformations to form strip shapes, flattened and near-spherical shapes. Meanwhile, Zn-6Cu particles were slightly oxidized to form ZnO resulting in higher oxygen content on particle boundaries than the particle inside as shown in Figure 3a. Although the Zn-6Cu deposits were heated at different conditions, the temperature was lower than the melting point of the Zn-6Cu. Therefore, there was no visible difference in the shapes of Zn-6Cu particles in deposits before and after heat treatment.

Inter-particle boundaries show some differences before and after heat treatment. In Figure 3a, inter-particle boundaries between the neighboring Zn-6Cu particles were clear and visible in the as-sprayed deposit. The deposits after heating at 200 °C exhibited similar features to the as-sprayed deposit as shown in Figure 3b. As the heating temperature increased, atomic diffusion became more intensive and the inter-particle interfaces could be completely disappeared [48,49]. Shown using purple arrows in Figure 3c,d, some inter-particle boundaries were discontinuous and invisible due to the coalescence as the heat treatment was increased up to 300 °C.

It can be found that both the pore size and porosity of Zn-Cu deposits were increased after heat treatment in Figure 3. The porosity was 0.12%, 0.23%, 0.56%, and 0.98% for the as-sprayed, Type-A, Type-B, and Type-C deposits, respectively. The increase in porosity was consistent with many reports about the effect of heat treatment on cold-sprayed metal deposits [48]. The Zn-Cu particles impact the substrate with high velocities and exhibit severe plastic deformations, and the adiabatic shear instability was mainly considered as a bonding mode of inter-particles in cold-sprayed deposits [37,52]. There were lots of tiny non-bonding regions between inter-particles in the as-sprayed deposits. During post heat treatment, these tiny non-bonding regions could be coalesced by atomic diffusion to form pores [48]. Therefore, although the inter-particle bonding was enhanced, the pores were formed and increased by heat treatment.

### 3.3. The Tensile Properties of Zn-6Cu Deposits Before and After Heat Treatment

**Figure 4:**
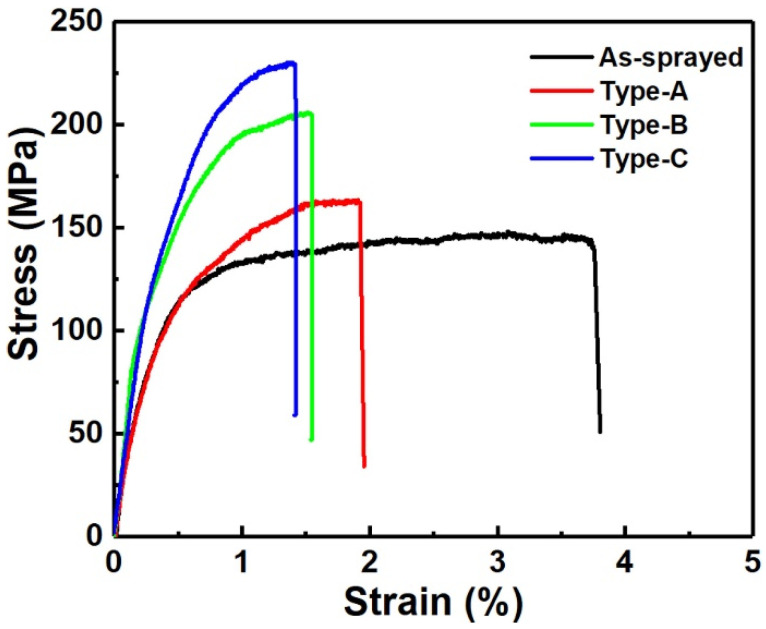
Tensile stress–strain curves of Zn-6Cu deposits before and after heat treatment.

**Figure 5:**
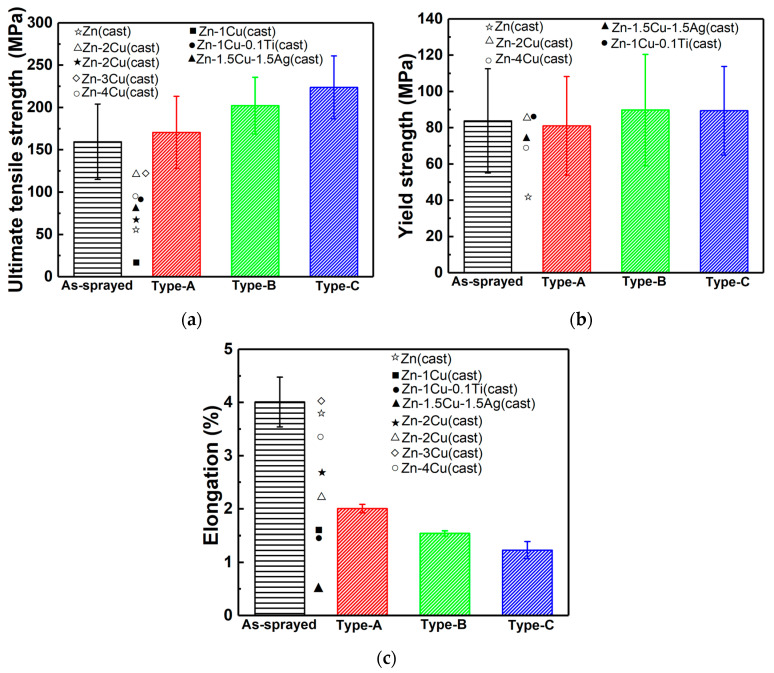
UTS, YS, and EL of Zn-6Cu deposits before and after heat treatment. (**a**) UTS; (**b**) YS; (**c**) EL. ● [19], ■ [50], ★ [50], ◇ [50], ✩ [55], ○ [56], ▲ [57], △ [58].

Figure 4 shows stress–strain curves of Zn-6Cu deposits before and after heat treatment and the relative mechanical properties are presented in Figure 5. It can be found that the yield strength (YS), ultimate tensile strength (UTS), and elongation (EL) of the as-sprayed Zn-6Cu deposit were 83.8 ± 28.7 MPa, 159.6 ± 44.5 MPa, and 4.1 ± 0.5%, respectively. After heat treatment of 200 °C, the YS and UTS of Type-A were, respectively, increased to 81.4 ± 27.2 MPa and 170.8 ± 42.6 MPa, but the EL was decreased to 2.1 ± 0.2%. After heat treatment of 300 °C, the YS and UTS of Type-B were, respectively, increased to 89.7 ± 30.7 MPa and 202.3 ± 33.5 MPa, but the EL was decreased to 1.5 ± 0.1%. The YS and UTS of Type-C were, respectively, increased to 89.4 ± 24.4 MPa and 223.8 ± 37.1 MPa, but the EL was decreased to 1.2 ± 0.2%. As shown in Figure 3, the inter-particle bonding in deposits after heat treatment was enhanced due to the atomic diffusion. The improvements of both YS and UTS of cold-sprayed Zn-6Cu deposits reflected the enhancement of inter-particle bonding [47,48,49,53,54]. However, coarse grains and increased pores could act as cracking resources during tensile testing, resulting in the decrease in elongation. The large standard deviations in YS and UTS could be attributed to the small sample size of MFT and the randomly distributed pores in the deposits. It is reported that the cast pure Zn shows a YS of 45 ± 3.5 MPa, a UTS of 61 ± 3.7 MPa, and a EL of 3.8 ± 0.8% [52]. Some cast Zn-Cu alloys shows the YS lower than 100 MPa, the UTS lower than 150 MPa, and the EL lower than 4%, as shown in Figure 5. However, the cast Zn-Cu alloys after severe plastic deformations exhibit significant improvements in YS, UTS, and EL [3]. Therefore, although the mechanical properties of the cold-sprayed Zn-6Cu alloy deposits were much lower than the counterparts after severe plastic deformations [3,55], it was comparable to and much higher than the cast pure Zn and Zn-Cu alloys [56,57,58]. Compared to the Zn-Cu alloys prepared by laser powder bed fusion additive manufacturing [59], the cold-sprayed Zn-Cu deposits exhibited higher tensile strengths and elongations. This result indicates that the potential applications of cold-sprayed Zn-6Cu deposits are comparable to the casting ones.

### 3.4. The Fracture Morphology of Zn-6Cu Deposits After Tensile Testing

**Figure 6:**
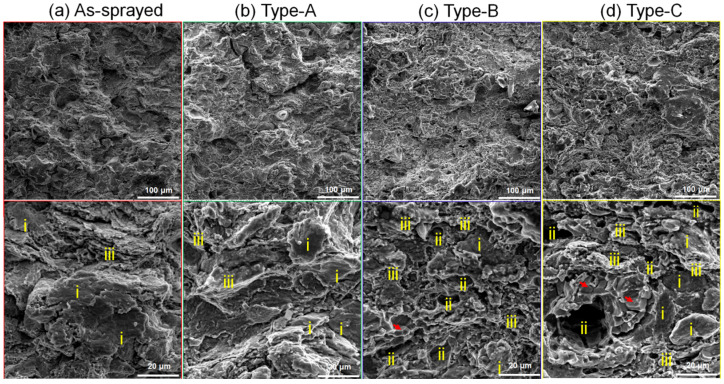
The fracture morphology of Zn-6Cu deposits after tensile testing. i: particle profile; ii: pores; iii: dimples; red arrows: cleaving particles.

To investigate the tensile behavior, the fracture morphology of Zn-6Cu deposits of different conditions after tensile testing was characterized in Figure 6. In the as-sprayed Zn-6Cu deposit, some tiny dimples and clear particle profiles with flattening were observed in Figure 6a. This was a typical fracture feature in cold-sprayed metals [37,49,52], which shows inter-particle bonding due to plastic deformations of metal particles. After heating at 200 °C, some dimples were observed in addition to the clear particle profiles in Figure 6b. As the temperature was increased up to 300 °C, the dimples indicative of trans-particle fracture were increased in Figure 6c,d. Cleaving particles and pores were also observed in Type-B and Type-C deposits. According to many studies [37,49,52], the increase in dimples indicates the improvement of the ductility. However, some large pores were observed and Zn grains grew in the Zn-Cu deposits at heating temperatures up to 300 °C as shown in Figure 6c,d. The pores could cause preferential stress concentrations along inter-particle boundaries [47,48,49,50,51,52,54], and the coarse grains could decrease the ductility of the deposits. Thus, the fracture of Zn-6Cu deposits shows a brittle rupture after heat treatment. It is reported that hot rolling and annealing could result in the densification and the refinement of the cold-sprayed metals and the elongation is improved [60]. This indicates that in the future the tensile strength and the elongation of cold-sprayed Zn-6Cu deposits could be further improved by combining with a post spray plastic deformation.

### 3.5. Friction and Wear Behavior of Zn-6Cu Deposits Under Dry and SBF Conditions

**Figure 7:**
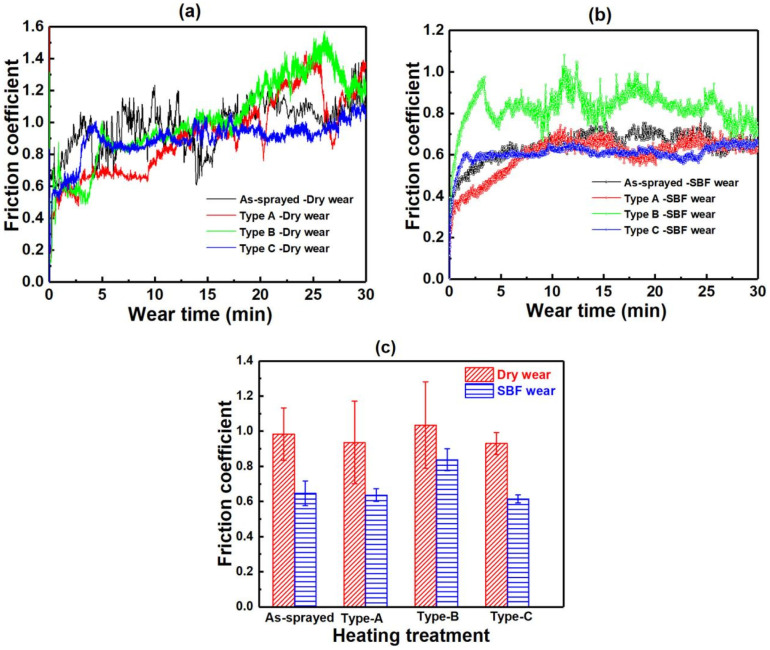
Friction coefficient curves of Zn-6Cu deposits before and after heat treatment. (**a**) Dry wear, (**b**) SBF wear, and (**c**) the friction coefficient.

Figure 7 shows the friction coefficient curves of the cold-sprayed Zn-6Cu deposits before and after heat treatment. In Figure 7a, it can be found that the friction coefficient of the Zn-6Cu deposits under dry wear was stable at the initial stage and then increased and sharply fluctuated with increasing wear time [19,21]. Figure 7b shows the friction coefficient of the Zn-6Cu deposits under SBF wear was relatively stable after the initial wear stage. Figure 7c shows the friction coefficient of all the samples fluctuated between 0.93 ± 0.06 and 1.11 ± 0.12, and the friction coefficient of SBF wear was lower than that of dry wear regardless of post heat treatment. Compared to the as-sprayed deposit, the friction coefficient of deposits after heat treatment was slightly lower in addition to Type-B.

**Figure 8:**
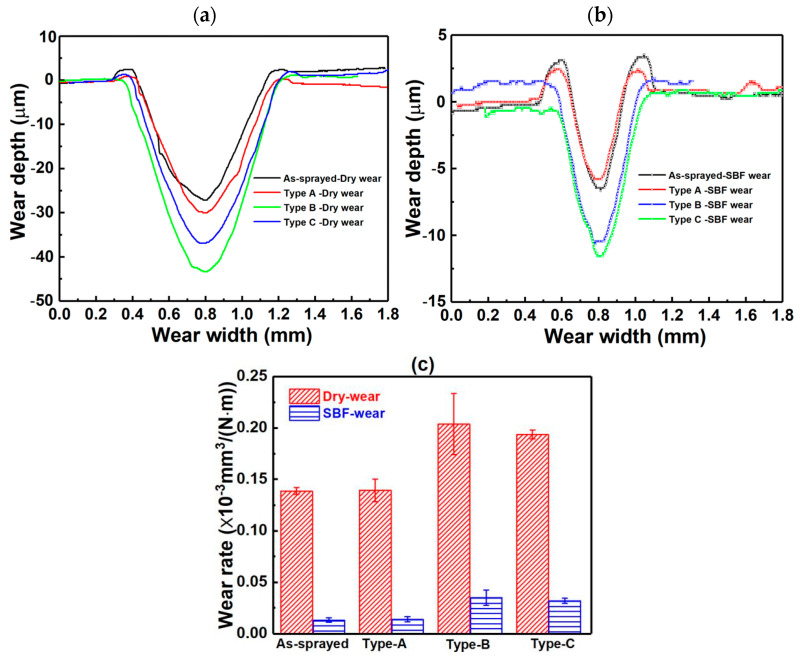
Wear profile of Zn-6Cu deposits before and after heat treatment. (**a**) Dry wear, (**b**) SBF wear, and (**c**) wear rate.

Figure 8a,b shows worn profiles of Zn-6Cu deposits before and after heat treatment. It can be found that the width and depth of worn surfaces were increased after heat treatment and the wear rate was also increased after heat treatment. As shown in Figure 8c, the wear rates of dry wear for the as-sprayed, Type-A, Type-B, and Type-C deposits were 0.139 ± 0.003 × 10^−3^, 0.139 ± 0.011 × 10^−3^, 0.204 ± 0.029 × 10^−3^, and 0.193 ± 0.004 × 10^−3^ mm^3^/(N·m), respectively. The wear rates of SBF wear for the as-sprayed, Type-A, Type-B, and Type-C deposits were 0.013 ± 0.002 × 10^−3^, 0.014 ± 0.002 × 10^−3^, 0.035 ± 0.007 × 10^−3^ and 0.032 ± 0.003 × 10^−3^ mm^3^/(N·m), respectively. It can be found that the anti-wear of cold-sprayed Zn-6Cu deposits was decreased after the heat treatment.

**Figure 9:**
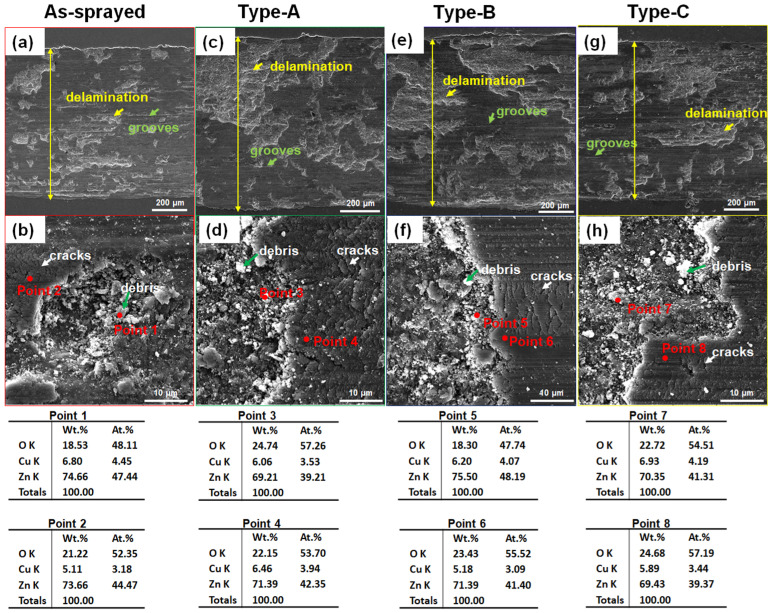
The worn morphology of Zn-6Cu deposits under dry wear. (**a**,**b**) As-sprayed, (**c**,**d**) Type-A, (**e**,**f**) Type-B, and (**g**,**h**) Type-C. The yellow double arrow line indicates the width of the wear scar.

**Figure 10:**
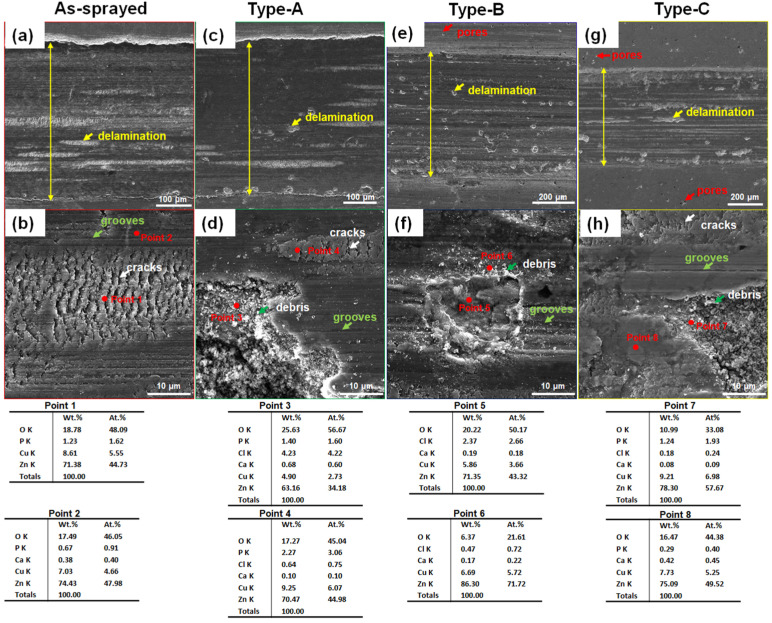
The worn morphology of Zn-6Cu deposits under SBF wear. (**a**,**b**) As-sprayed, (**c**,**d**) Type-A, (**e**,**f**) Type-B, and (**g**,**h**) Type-C.

Figure 9 shows the worn surface morphology of different Zn-6Cu deposits after dry wear. In Figure 9a,b, shallow grooves and small localized delamination were the main wear features. The localized delamination was enlarged after heat treatment as shown in Figure 9c–h. There were some debris on the delamination regions and some cracks were observed on the grooves. Comparing with original deposits, oxygen content of both the debris and the grooves was increased after wear as shown in Figure 3. It can be found that the localized delamination was the main wear feature for dry wear rather than the groove. In Figure 10, both the grooves and delamination were also observed after SBF wear. The localized delamination was also increased after post heat treatment as shown in Figure 10c–h. However, the quantity and the size of localized delamination were significantly lower than that under dry wear. The oxygen content of worn surfaces under SBF wear was slightly lower than that under dry wear. In addition to O, Cu and Zn, Cl, P, Ca atoms were also detected on the worn surface after SBF wear, which was the residue from SBF. Comparing with the dry wear, the groove was the main wear feature for SBF wear.

**Figure 11:**
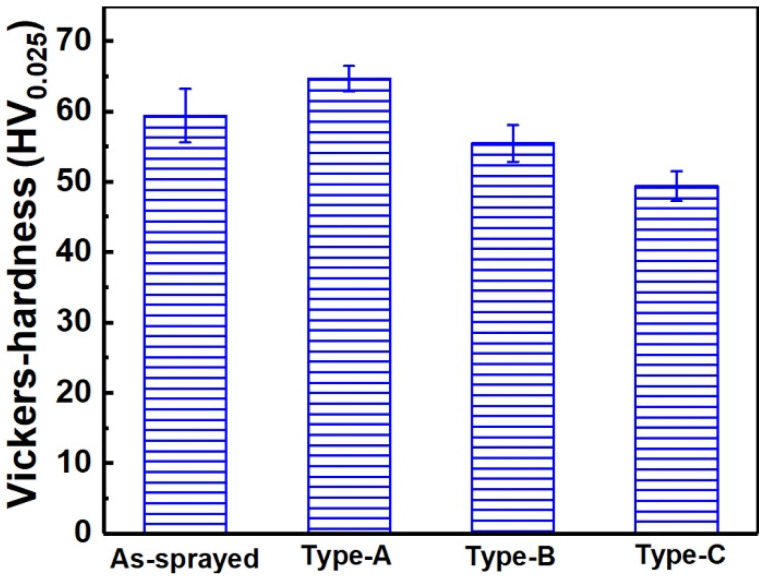
Microhardness of Zn-6Cu deposits before and after heat treatment.

During sliding wear, the Si_3_N_4_ ball was in contact with and experienced wear against the Zn-6Cu deposit. The reciprocating friction could not only cut the sample to form grooves and delamination, but also increase the temperature [61,62]. During cold spraying, Zn-6Cu deposits were formed by particle accumulations with serious plastic deformations. The pores and weak inter-particle boundaries in deposits acted as delamination sources during wear. Particles were peeled off from the deposits to form large localized delamination during reciprocating wear. The decrease in microhardness of Zn-6Cu deposits after heat treatment was shown in Figure 11, which could increase the worn width and the worn depth after heat treatment. Although the inter-particle bonding was enhanced after heat treatment, the coarse grains and the increased pores could result in the increase in the localized delamination. Some delamination and debris on the worn surface further could act as abrasive particles under dry wear [19,63,64]. Thus, this is a type of three-body wear for dry wear and its main wear feature was the localized delamination. Comparing with dry wear, the SBF can act as a lubricant and the friction coefficient was lower. The localized delamination under SBF wear was lower and smaller than that under dry wear. Furthermore, the delamination and debris on the worn surface were also washed away from the wear tracks by the SBF [19,65]. And the SBF could take away the heat generated by the friction process over time. Even if the deposits were worn in the stimulated body fluid, the oxygen content on the worn surface was still lower than that of under dry wear. The wear occurred between the worn pair and the deposits during SBF wear, and the main wear feature was the groove. As well known, the implant materials are inevitably in contact with and wear against tissues during serving in vivo. Therefore, this low wear rate of SBF wear for cold-sprayed Zn-6Cu deposits presents a meaningful potential application in implants.

## 4. Conclusions

Zn-6Cu alloy was successfully prepared by cold-spraying additive manufacturing technology, and the effect of post heat treatment on microstructure, tensile and wear behavior was investigated. Cold-sprayed Zn-6Cu deposits exhibited a dense structure and flattening particles, and both the porosity and inter-particle bonding were increased after heat treatment. Cold-sprayed Zn-6Cu deposits before and after heat treatment were composed of Zn and CuZn_5_ phase in addition to a little ZnO. The yield strength and ultimate tensile strength of Zn-6Cu deposits after post heat treatment were, respectively, increased from 83.8 ± 28.7 MPa and 159.6 ± 44.5 MPa to 89.4 ± 24.4 MPa and 223.8 ± 37.1 MPa. The fracture morphology of Zn-6Cu deposits after heat treatment exhibited brittle features in addition to dimples. The Zn-6Cu deposits before and after heat treatment exhibited comparable friction coefficients. The wear rates of dry wear for the as-sprayed, Type-A, Type-B, and Type-C deposits were 0.139 ± 0.003 × 10^−3^, 0.139 ± 0.011 × 10^−3^, 0.204 ± 0.029 × 10^−3^, and 0.193 ± 0.004 × 10^−3^ mm^3^/(N·m), respectively. The wear rates of SBF wear for the as-sprayed, Type-A, Type-B, and Type-C deposits were 0.013 ± 0.002 × 10^−3^, 0.014 ± 0.002 × 10^−3^, 0.035 ± 0.007 × 10^−3^ and 0.032 ± 0.003 × 10^−3^ mm^3^/(N·m), respectively. The decrease in the wear rate of SBF wear was mainly attributed to the lubricant of simulated body fluid. Zn-6Cu deposits before and after heat treatment exhibited the same wear features of grooves and localized delamination regardless of the wear condition.

## Data Availability

The raw data supporting the conclusions of this article will be made available by the authors on request.
